# A Comparative Study on Different Pharmaceutical Industries and Proposing a Model for the Context of Iran

**Published:** 2018

**Authors:** Hossein Safari, Mohammad Arab, Arash Rashidian, Abbas Kebriaee-Zadeh, Hasan Abolghasem Gorji

**Affiliations:** a *Department of Health Management and Economics, School of Health, Tehran University of Medical Sciences, Tehran, Iran. *; b *Department of Toxicology and Pharmacology, School of Pharmacy, Tehran University of Medical Sciences, Tehran, Iran. *; c *Department of Health Services Management, School of Health Management and Information Sciences, Iran University of Medical Sciences, Tehran, Iran.*

**Keywords:** Pharmaceutical industry, Growth and development policies, Health, Qualitative study, Iran

## Abstract

Medication is known as the main and the most effective factor in improving public health. On the other hand, having a strong and effective pharmaceutical industry will, to a very large extent, guarantee people′s health. Therefore, this study was prospected to review the different pharmaceutical industries around the world and propose a model for the context of Iran. This is a qualitative as well as a comparative study which was carried out in 2015. At the first stage, using the World Bank website, countries were divided into four groups of low-income, lower-middle-income, upper-middle-income, and high-income economies. Then, four countries of Afghanistan, India, Brazil, and Canada were chosen from these four groups, respectively. Secondly, data gathered from these countries were given to two 12-member expert panels. Finally, using the articles and the results of expert panel groups, useful and effective policies were extracted for the growth and development of Iran′s pharmaceutical industry. Findings of the study indicated that the following seven items are the essential policies for the context of Iran: establishment of high academic centers as well as research institutes, using weak patent law, supporting research and development centers at universities and pharmaceutical companies, backing national pharmaceutical companies up, implementing generic rules, gradual economic liberalization, and membership in world trade organization. Since, pharmaceutical industry is an effective and inseparable part of every health system, proper and evidence-based policies should be taken into account in order to develop this industry and, ultimately, meet the public needs.

## Introduction

 Health has turned into one of the most substantial issues in different communities and this has increased the demands for health care services. Due to these high demands, most countries have faced with increased health expenditures. Pharmaceutical industry is no exception in this regard, and it is one of the areas which has played a vital role in increasing the health expenditures (1, 2). On the other hand, medication is known as the main and the most effective factor in improving public health as well as in controlling some diseases among people (3).

 Considering the importance of public health, governments have implemented some strict and fundamental rules for health–related industries, so they can improve public health through controlling the costs and, therefore, can provide the grounds for development of such industries including pharmaceutical industries. Development carries a qualitative meaning and is a process through which fundamental changes and reforms take place at economic, social, cultural, and political levels. This can result in creation of new production methods and a shift from traditional to modern ways of improving industries (4, 5). 

 Moreover, improving public health and developing the pharmaceutical industry are the two most important challenges countries face around the world. Pharmaceutical industry –as one of the most vital parts of any health system- is strictly monitored and controlled by governments. Implementation of some strict rules as well as microscopic supervision of governments on pharmaceutical companies have considerably affected their development and growth. Any wrong selection of rules or policies may result in finishing up the life of many pharmaceutical companies which itself will risk the public health. On the other hand, implementation of proper policies will help this industry grow and develop, but it will also assure the availability of drugs at the right place, with the right price and quality, and, ultimately, will improve the community's health. Meanwhile, pharmaceutical companies should try to adapt themselves with these policies in order to, firstly, maintain their status quo, and, secondly, create development and growth through turning threats into opportunities or even using the current available opportunities (6, 7).

 Governments and policy makers of countries around the world are well aware that development and growth of national industries will result in economic growth along with creation of job opportunities, wealth production, poverty reduction, and increased trades with other countries, and may also bring about high technologies to the national pharmaceutical industry. If pharmaceutical industry-as one of the important industries- witnesses considerable and dramatic growth, then, essential as well as non-essential drugs will be provided for the community with high quality and at the right price. So, people will benefit from such growth, and this will improve the community's health, and we will have healthier people at work (8, 9). 

 Considering the high advancements in the medical area, life expectancy of people in developing and developed countries is rising up, which it will result in more aged population in coming years. These people will definitely require some drugs, therefore, we will witness the enlargement of pharmaceutical markets in near future (10, 11). With regards to the above-mentioned points, this study was prospected to present a proper model for development and growth of Iran's pharmaceutical industry.

## Experimental

 This is a qualitative and comparative study which was carried out in 2015. Since the researchers wanted to study the pharmaceutical industries of the countries around the world. Therefore, it was not feasible to choose all countries. Therefore, it required a criterion so as to classify countries and select some countries randomly from those categories. Hence, after consulting with research supervisors, it was decided to use income level of countries- as a criterion affecting the growth and development of pharmaceutical industries- in order to classify the countries. Then, using the World Bank website, countries were divided into* four groups of low-income, lower-middle-income, upper-middle-income, and high-income economies. Then, four countries of Afghanistan, India, Brazil, and Canada were chosen from these four categories, respectively. Later on, Turkey- a country from upper-middle income category- was added to the study due to its progress in its pharmaceutical industry in the recent years. Next, *we went through the "Scopus," "PubMed," and "Google Scholar" databases using key words of "Pharmaceutical policy and growth" and "Pharmaceutical policy and development." 

We tried to select the most related articles which met the following criteria:

 Inclusion of pharmaceutical rules and policies as well as the role of governments in development and growth of pharmaceutical industry

 Having a time period mentioned for the rules, policies, and interventions

 Outlining the effects of pharmaceutical rules, policies, and interventions

 Clarifying the reason(s) behind implementation of these rules and policies

 The aim of this stage was to extract the rules and policies for the pharmaceutical development and growth of chosen countries as well as governments' roles in supporting their pharmaceutical industry. Then, these data were used to prepare the interview guide for the second stage. 

 In the second stage, twenty-four interviewees were invited to the expert panel discussions. They were experts in the pharmaceutical areas and were selected by the help of my supervisor, who has been working in the pharmaceutical area since Islamic Revolution. These experts were selected using the following criteria:

 Having management experience in pharmaceutical industry or related companies

 Having work experience in ministry of health

 Being familiar with international pharmaceutical markets

 Having work experience in research and development centers of pharmaceutical companies

 These twenty-four interviewees then were divided into two 12-member groups. This was done in order to prevent from any kind of crowdedness and to control the panel effectively. Pharmaceutical rules and policies gathered in the previous step were given to these experts and they were asked to express their opinions on the feasibility and appropriateness of each single policy. They were even asked to add the necessary points to the list of policies. Finally, using the articles and the results of expert panel groups, useful and effective policies were extracted for the growth and development of Iran's pharmaceutical industry.

## Results

Here, the conditions of the pharmaceutical industry in different countries are assessed and then the experts′ opinions have been used to shed light on Iran′s pharmaceutical industry. 

*Afghanistan*

The quantity of both donated and privately imported medicines entering Afghanistan has been considerably increased since 2002. Most part of the pharmaceutical market (70 to 80%) is owned by private sector and the market may worth up to US$200m per year. Afghanistan has a National Essential drug list which determines the medicines for use in public health facilities. There are some limitations imposed on privately imported medicines by ministry of health. One of the weaknesses of pharmaceutical industry in Afghanistan is the widespread smuggling of medicines into this country. Moreover, Afghanistan has a chaotic pharmaceutical market. Medicines are brought into the country from many diverse sources, and there is a puzzling array of products on sale. The number of actors is larger at every point in the supply chain than in other studied markets. There are more importers, more wholesalers, many more pharmacies, many grocery stores that sell medicines and street vendors of medicines as well as purveyors of traditional medicine (12, 13). There is some evidence showing that doctors may over-prescribe medicines without paying attention to the possible side effects. Patients often ask pharmacists to prescribe medicines even though a large proportion of private pharmacies do not have a qualified pharmacist on staff. What is worse is the presence of low quality and fake medicines containing insufficient ingredients on the market. Afghanistan also suffers from inadequate inspection, sampling, and testing facilities to meet the basic standards of medicines on the market. Given the scale of smuggling, it would be much better if efforts were concentrated on inspection and testing at the point of wholesale and retail. However, the absence of testing facilities at border points, long delays in clearing imports, and pending sample results from Kabul are serious impediments for importers to bring their imports through official channels. Recently, the government of Afghanistan has been working on having testing facilities installed at borders or even having mobile laboratory. In addition, there is not much control and regulation on pricing of pharmaceuticals in this country (14, 15).

*India*

With the implementation of patent law in 1911, all of the innovations including products as well as their production methods became patentable for 36 years. This law was a copy of patent law of Britain launched in 1852. This law resulted in creation of a free market for multi-national companies in a way that it made India import its pharmaceutical products from mother countries and they had no tendency to produce their own patented products within the country and did not even let its companies produce them (16). Another important point about India is the presence of common committees between ministries of health and science and technology. These committees are responsible for making arrangements between these two ministries so that they can set up proper and harmonized regulations for the growth and development of the national pharmaceutical industry. Moreover, Indian government announced a law for making its industries adapt themselves to trade related aspects of intellectual property rights (TRIPS). According to this law, there was no patent right for new applications of the old drugs, no patent for the mixed drugs, and no patent for derivatives of a new molecule if it has not increased the effectiveness of the previous molecule (17, 18).

Indian government not also tried to harmonize their pharmaceutical rules with TRIPS, but they also intelligently used the flexibilities of TRIPS to create suitable conditions for growth and development of its pharmaceutical industry. Through this, they also protected their national companies from harms of sever and unfair international policies and rules. Therefore, Indian companies could follow different paths such as producing generic drugs, investment in research and development departments so as to produce new drugs, partnership with multinational companies in research areas, marketing their patented drugs and producing them through signing contracts with their owners (19).

India has also implemented some rules for research and development of their companies. These rules include giving tax exemptions up to 150% for those investments in R and D sections as well as for those companies which use national technologies; government′s financial support of common research projects between universities, research centers, and pharmaceutical companies since 2004; providing low-interest rate loans for pharmaceutical companies; and giving a 5-year tax exemption to those companies involved in research and scientific projects (17, 20).

*Brazil*

It was after mid-20^th ^century that both India and Brazil decided to bring growth and development to their national industries, especially to the pharmaceutical industry. Both countries tried hard to implement weak patent laws for a certain time and to create a big national market along with training many scientists and experts. In 1930, Brazil′s pharmaceutical market included some research institutes, national pharmaceutical companies as well as some multi-national companies which held 13.6% of Brazil′s pharmaceutical market (21). In 1990, federal government of Brazil passed on a law according to which establishment and development of companies required a formal license from the government. The aim of this law was to encourage investments in building or developing companies in strategic industries, and to reduce the imports and any kind of dependence on foreign countries (22). In recent years, Brazil has tried to improve the collaborations between pharmaceutical companies and universities so as to enhance research activities and use their results for development of pharmaceutical industry (23). In addition, those companies which are active in research and development areas can benefit from income tax exemptions or can be financially supported for buying new and essential equipment (24). Brazil has also built some research centers for producing essential drugs for diseases such as AIDS so that they could reduce their dependence on importing expensive and high-tech drugs for such diseases. Moreover, they have put some custom tolls and duties on importing any drug which is being produced by national companies. Finally, they passed on the generic law in 1999 in order to support national pharmaceutical companies and to make the drugs affordable and accessible to the public. This law was also concerned with packaging, marketing, and promotion of drugs (21). 

*Canada*

Due to high prices of drugs in 1960 and in order to help the pharmaceutical industry grow, government of Canada authorized a patent law according to which patent duration changed to 17 years. This law also gave the patent owners the permission of using compulsory licensing for both imported and nationally produced drugs provided that they have paid 9% of the pure price of drug selling. This licensing reduced the governmental costs up to $212 million in 1983 (25). In 2004, a bill called C-9 or C-56 was passed on according to which government of Canada, with respect to the act 2003 of TRIPS, allowed companies to produce and export their patented drugs to under-developed or developing countries. This bill was authorized for two years in the beginning but was extended after that (26). Canada also passed on a law to support their national companies and their importing. According to this law, those drugs which were produced only for importing did not require any registration inside the country and should only be certified and registered in the target country. Of other supports of government from national companies was replacement of drugs by their generic forms which was later called «generic substitution». Moreover, they offered some tax exemptions for those actions aiming at development of research and production of new drugs in 1983 (27, 28).

*Turkey*

Pharmaceutical industry of Turkey has had, on average, a 14% growth from 1995 to 2000. This was much bigger than the 7.2% growth of European countries at the same time. One of the weaknesses of Turkey′s pharmaceutical industry is that it takes 2.5 years for a company to get a license for producing a generic drug. On the other hand, its controlling system for drug prices has stopped this strategic industry from development. In Turkey, most researches are done by universities but they are of no use since there are not strong and big private pharmaceutical companies to use the results of such researches. This is considered as one of the reasons behind low investment in R and D sections (29, 30). 

Since 1961 and because of essential drugs being expensive along with Turkey′s market turning into a monopolistic market for multi-national companies, they called off patenting any product as well as the production methods of drugs, and this led to a dramatic growth among national companies (29).

In 1999, Turkey signed a contract with World Trade Organization (WTO) according to which it was supposed to include drug patents and production methods in its rules. Turkey tried to control the imports so as to support the national producers and provided subsidies for pharmaceutical companies as well. On the other hand, national companies gained a lot of profit through importing raw material and working only on finished products. Furthermore, in order to support researches within the country, they prevented foreign companies from producing the finished products and these companies should start from the first levels of drug production if they want any production license (18). 

*Experts′ opinions on Iran′s pharmaceutical industry*

*Obstacles ahead of growth and development of pharmaceutical industry in Iran*

Presence of some infrastructural problems such as lack of a proper and suitable transportation system for development of industry, lack of high speed and reliable information and communication networks as well as weak banking system for facilitating international exchanges have created some obstacles for national pharmaceutical industry. Inconsistency of monetary and production policies along with instability of top managers′ positions are among the other issues facing the pharmaceutical industry in Iran.

*Government′s role in growth and development of pharmaceutical industry*

Since most of the pharmaceutical companies in Iran are owned by government and it is the main drug buyer (through insurance companies) as well, therefore, not only they can play an important role in implementation of national and international policies for development of pharmaceutical industry, but also can establish exact, feasible, and comprehensive plans to meet those regulations and policies. Macro-policies of government determine the pharmaceutical regulations and policies at micro level. Iranian government can pave the ground for growth and development of pharmaceutical industry through putting some limitations on importing drugs, exempting some companies from paying customs toll and duties, provision of tax exemptions and low-interest rate loans for those research-centered companies. Although government plays the most fundamental role in pharmaceutical industry, some experts believe that privatization is what Iranian pharmaceutical industry needs. 

In order to support national companies and prevent from wasting capitals of insurance companies, government can differentiate essential and strategic drugs from other products, modify insurance policies (such as leaving OTC drugs out of insurance list and putting national products on that list), adjust drug prices along with putting some incentive prices, and alter prescribing behaviors of doctors and encourage them to prescribe generic drugs.

*Doctors′ role in growth and development of pharmaceutical industry*

Physicians do not usually trust national pharmaceutical products in Iran, so they go for prescribing foreign drugs without even being aware of their quality. Some nationally produced drugs have even more quality than those imported ones. Doctors should be totally informed of these drugs through holding some conferences and seminars. 

As seen in Table 1, factors such as inconsistency in management, industrial, production, and monetary policies; lack of targeted planning and support for university level researches as well as the weak relation between university and industry; and absence of support from R and D sections of national companies are among the main obstacles facing pharmaceutical industry in Iran.

**Table 1:** Obstacles facing pharmaceutical industry in Iran

	**Obstacle**	**No. of experts agreed on it**	**Percentage**
1	Involvement of Iran in international political challenges and the results of imposed sanctions	12	54.5
2	Inconsistency in management, industrial, production, and monetary policies	17	77.2
3	Inflation and banking system weaknesses	10	45.5
4	Ignoring joint venture among small-size companies	14	63.6
5	lack of targeted planning and support for university level researches as well as the weak relation between university and industry	16	72.7
6	Absence of support for R and D sections of national companies	15	68.1
7	Lack of support from national pharmaceutical companies	13	59
8	Lack of information about quality of nationally produced drugs as well as brand prescription behaviors among doctors	14	63.6
9	Ownership of most pharmaceutical companies by government	15	68.1
10	Weak and broken insurance policies	16	72.7

**Figure 1:**
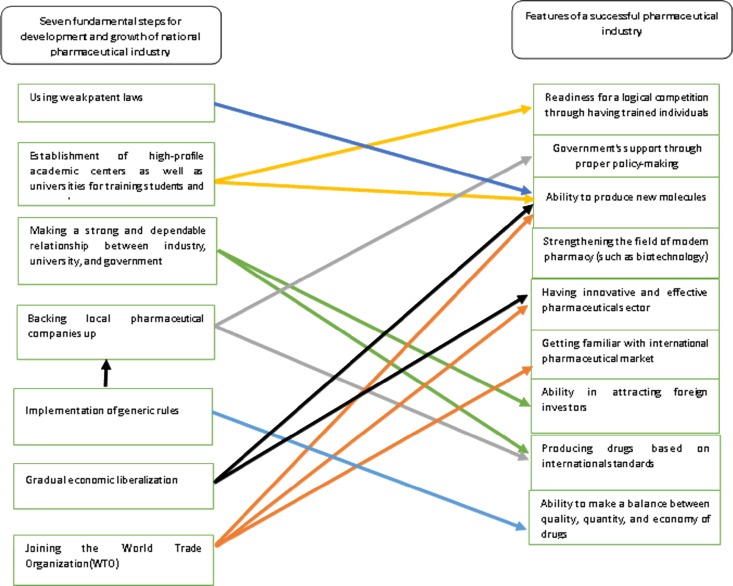
Conceptual framework for development and growth of Iran's pharmaceutical industry

Ultimately, using the above-mentioned information and considering the features of a successful pharmaceutical industry, a framework was developed indicating how Iran′s pharmaceutical industry can use these steps in order to improve its conditions (Figure 1).

## Discussion

The current study was prospected to present a proper model for development and growth of Iran′s pharmaceutical industry. Having searched articles along with expert panel interviews meticulously reviewed, seven most important and fundamental steps for development and growth of national pharmaceutical industry were derived. These steps involve the following:

*1-Establishment of high-profile academic centers as well as universities for training students and researchers in pharmacy, medicine, chemistry, and related majors in order to work in pharmaceutical industry or at the research and development (R and D) centers*

This accounts for as one of the most fundamental policies for growth and development of pharmaceutical industries. Universities and high profile academic centers should best use their facilities for improving the scientific and technical capabilities of pharmacy students and researchers and prepare them for R and D laboratories at universities and pharmaceutical companies as well as for working in production and managerial sections of pharmaceutical companies. 

Although Iran has many universities for training students in pharmacy, medicine, chemistry, and other related fields of study, the results of researches are not properly used for producing new and innovative products. Moreover, there are some weaknesses in practical aspects of university curricula as they are not properly and usefully implemented in the country. On the other hand, lack of growth and development of pharmaceutical companies, especially in the area of R and D which is mainly focused on drug formulation aspects, has reduced the need for professional researchers in the companies and made them leave the country to conduct those researches somewhere else. It seems that with putting more attention on practical courses as well as apprenticeship of students along with equipping universities with facilities for R and D and reforming supportive policies and rules for researches, the needed motivation and drive in our researchers so as to produce new and innovative products can be achieved. Furthermore, the relationship between government, industry, university, and research centers should be enhanced. Financial and non-financial supports by government can pave the ground for presence of university professors and researchers in pharmaceutical companies and help the country use the highest potentials of such groups for national growth and development.

*2-Using weak patent law in order to develop the reverse engineering and increase the capabilities of pharmaceutical companies for conducting fundamental researches and, ultimately, producing brand products*

As mentioned earlier, using weak patent law for developing reverse engineering, increasing the capabilities of national companies in producing pharmaceutical products as well as learning the development trend of a drug are the basic steps adapted in many countries. In this regard, India from 1972 to 2005, Brazil from 1945 to 1969, and China from 1985 to 1992 patented only the pharmaceutical production method. Moreover, pharmaceutical products were not patentable before 1949 in England, 1960 in France, 1958 in Germany, and before 1978 in Swiss, Sweden, and Italy. Using weak patent law, especially in the area of pharmaceutical production method, has increased the abilities of these countries′ pharmaceutical companies in producing new drugs. Of course, we can′t overlook the governmental supports in these countries in providing a suitable environment for growth and development of their pharmaceutical industry. Endorsement and implementation of weak patent law are among the macro-policies of Iranian government. It seems that Iran has so far been able to use these rules properly, but there have been no long-run and targeted plans for achieving over the borders goals. Inter-ministry committees should be arranged in order to find proper and feasible solutions for problems of industries, especially pharmaceutical industry, in Iran. 

*3-Supporting R and D centers at universities and pharmaceutical companies and making a strong and dependable relationship between industry, university, and government*

Governments have been supporting pharmaceutical research actions mainly through the following:

Financial supports from researches done at universities, research centers, and R and D departments of pharmaceutical companies

Financial supports through giving low-interest rate loans for importing supplies and consumables for conducting the researches

Financial supports in form of tax and duty exemptions for importing supplies and consumables for conducting the researches

Tax exemptions for those researches resulting in production of a new product or new production method

Offering price incentives for those researches resulting in production of a new product or new production method

Due to high costs of producing raw pharmaceutical material, low marginal profits, and lack of governmental supports, private pharmaceutical companies can′t properly invest in R and D sections. Despite the advancements in reverse engineering, unfortunately, most of the activities in R and D departments are focused on formulation of drugs. Government should provide more supports for companies involved in research actions. On the other hand, most of the supports are shifted towards researches resulting in article publication, but they should support those researches which will lead to a new product. 

*4-Backing local pharmaceutical companies up so as to enhance their capabilities in producing pharmaceutical raw materials, meeting national pharmaceutical needs, and increasing their competitive features against multi-national companies*

Not only we need to enact some policies in order to support R and D departments of pharmaceutical companies, but we also need to back up the companies so that they can compete with multi-national companies through updating their equipment and technologies. Most of the developed and developing countries apply the following procedures to support their pharmaceutical companies:

Placing high customs toll and duties on importing foreign goods

Implementing tax exemptions

Considering some subsidies for companies

Assigning the monopoly rights of producing some goods to some small-size companies 

Implementation of some policies for making foreign companies produce the pharmaceutical raw material as well as some drugs inside the country 

Providing low-interest rate loans for companies in order to help them update their equipment and adapt themselves with good manufacturing practice (GMP) procedures

Making joint-ventures among small-size companies with an aim of creating bigger and stronger companies 

Applying the rules of TRIPS so as to develop and grow the national industry 

Due to involvement of Iran in some international political challenges, especially those sanctions that have been imposed on Iran after Islamic revolution, there have been some obstacles for businesses within the country; therefore, these businesses require more governmental supports for their strategic growth and development. On the other hand, the presences of high inflation rate along with broken banking system also prevent the growth and development of the national industries. Moreover, inconsistency of rules and regulations along with successive changes of management positions do not allow industries to form steady programs for their development. These changes have also broken the dependable trust among universities, industries, and government. Government can play a vital role in development and growth of pharmaceutical industry through bringing stability to production and monetary policies. 

*5-Implementing generic rules in order to support national generic drugs producers*

Generics act is usually supposed to support national companies that produce generic drugs, back up the patient rights, and prevent from wasting the capitals of insurance companies. Although this act has been active in Iran since the Islamic revolution, it owns some weaknesses as well. 

Buying generic drugs by government, making pharmaceutical companies imprint the generic name on their products as well as making doctors, who work in the governmental sectors, prescribe generic drugs for patients are among the important actions taking place under the name of generic act in Iran. Unfortunately, Iranian doctors are not quite aware of the quality of national pharmaceutical products. For this reason, they do not tend to prescribe generic drugs for their patients, and this has brought some dramatic costs to health and treatment sectors. 

*6-Gradual economic liberalization for developing and enhancing pharmaceutical industry when they are equipped with competitive capabilities*

Many of developing and developed countries take on a gradual movement towards economic liberalization when they reach a reasonable level of growth and development among their national industries in a way that they are capable enough to compete with their international competitors. Of the influential actions taken to reach this goal are reducing customs toll and duties for importing foreign drugs, privatization of governmental companies, and reducing the number of drugs under the control of pricing system (22). Iran has many insurance companies which are the main drug buyers. On the other hand, some of the very strong and huge pharmaceutical companies severely control the pricing of drugs in Iran. The pricing system of drugs in Iran does not correspond to the efforts of companies, so they are not willing to invest in producing innovative drugs or in their R and D departments. Using some encouraging pricing may move companies towards innovation, research and development actions. 

Moreover, non-privatization of governmental companies or even non-development of them is a big obstacle on the way of growth and development of pharmaceutical industry in Iran. 

*7-Joining World Trade Organization (WTO) and compliance with all patent laws of TRIPS*

Countries like Turkey, China, Canada, Brazil, and India have harmonized their industrial and monetary policies with WTO by the help of gradual economic liberalization; therefore, they have supplied more of their pharmaceutical products, especially generic ones, to the western developed countries. They have also enlarged their market share and, through this, are investing more in producing innovative and high-tech drugs. On the other hand, patients in these countries enjoy their accessibility to new foreign drugs because of their countries′ membership in WTO. India is one of those countries that has used TRIPS to adapt itself with the rules and policies of WTO, and its pharmaceutical companies have found their way in the world market (16). Although Iranian government has shown no willingness towards joining WTO, they should use the flexibilities of TRIPS for development and growth of its pharmaceutical industry. Perhaps, one of the most important issues in joining WTO involves reduction of tariffs or even immediate elimination of them. Because, implementation of tariffs and tolls will affect indexes and trade volume of Islamic republic of Iran in having business ties with the outside world. Although joining WTO will positively affect the export (through improving technical knowledge, having access to modern technologies...), import (through collaboration with international companies…), quality (through following international standards…), and survival of the national pharmaceutical industries (31), this should be taken into consideration that being a member of WTO requires proper infrastructure and appropriate management. Iranian government should adjust some of the business, monetary, and economic rules and policies so as to adapt itself with the requirements of WTO. Moreover, government should provide facilities for pharmaceutical companies in order to help them use modern technologies and improve their GMP.

## Conclusion 

These are the main steps which can be taken to bring about a change in Iran′s pharmaceutical industry. Along with these steps, there is a dire need to accurate and long-run planning, tax control and reforming the banking system, revision of insurance rules and policies, and changing doctors′ prescribing behavior. 
